# Identification and Potential Clinical Utility of the *MTNR1B* rs10830963 Core Gene Variant Associated to Endophenotypes in Gestational Diabetes Mellitus

**DOI:** 10.3389/fgene.2020.00332

**Published:** 2020-04-21

**Authors:** Gábor Firneisz, Klara Rosta, János Rigó, Ákos Nádasdi, Jürgen Harreiter, Alexandra Kautzky-Willer, Anikó Somogyi

**Affiliations:** ^1^2nd Department of Internal Medicine, Semmelweis University, Budapest, Hungary; ^2^MTA-SE Molecular Medicine Research Group, Hungarian Academy of Sciences - Semmelweis University, Budapest, Hungary; ^3^Department of Obstetrics and Gynecology, Medical University of Vienna, Vienna, Austria; ^4^1st Department of Obstetrics and Gynecology, Semmelweis University, Budapest, Hungary; ^5^Gender Medicine Unit, Division of Endocrinology and Metabolism, Department of Internal Medicine III, Medical University of Vienna, Vienna, Austria

**Keywords:** genotype, GDM, therapy, prediction, precision medicine, BMI, LGA

## Introduction

Genome-wide association studies (GWASs) are reliable tools to identify novel gene variant–trait (disease) associations. Recently high ranked journals published exciting reviews on the potential in the health predictions based on GWAS results (Tam et al., 2019). This indicates a renewed interest in the clinical use of polygenic risk and hazard scores (PRS and PHS) for prevalent diseases.

Despite the potential of PRS/PHS that can be applied to evidence the genetic component of specific clinical and treatment phenotypes is suggested to be substantial (Lambert et al., 2019; Loscalzo, 2019; Meigs, 2019; Srinivasan et al., 2019) we are not aware of any clinical praxis guideline in the diabetes mellitus (DM) field today that would have had been implemented to help clinical decisions based on GWAS findings.

This indicates a slow progression of clinical utilization of the genetic association study results. This slow transition to clinics is particularly contrasting when compared to standard diabetes care which is improved greatly due to the number of newly introduced glucose-lowering drugs and also to the novel follow-up/diagnostic techniques (Davies et al., 2018).

There is an enormous context gap between the everyday routine clinics and the 3,730 published (all traits) GWASs identified a total of 37,730 single nucleotide variants (SNVs) and 52,415 unique SNV–trait associations (till January, 2019) (Tam et al., 2019).

## Polygenic Risk and Hazard Scores

The most often proposed clinical use of complex genetic data obtained from GWAS is the development of polygenic risk or hazard scores (PRSs/PHSs) (Sarraju and Knowles, 2019). Genome-wide PRS and PHS scores are summed up of genetic risks associated to many individual independent genetic effects and developed to predict a clinical outcome, e.g., a development of a disease with polygenic background (Khera et al., 2018; Florez, 2019).

The literature outlined that the effect of individual gene variants on complex common disease phenotype is relatively weak and in these cases the PRS might be useful (Lambert et al., 2019; Loscalzo, 2019; Meigs, 2019; Srinivasan et al., 2019). Effect sizes of common genetic variants for T2DM are modest ever since the early GWAS era and are usually ranging from 10 to 35% (Lyssenko and Laakso, 2013). Consistently the OR is typically in between 1.1 and 1.3 and the OR for the identified common gene variants for T2DM remains below 1.7 (Prasad and Groop, 2015).

However, due to that GWASs require high sample sizes the studied prevalent disease eventually may be composed of largely heterogeneous subgroups (Ahlqvist et al., 2018). In addition as the sample sizes are increasing further novel rare and/or small effect variants might be found. The task is further complicated by social, population diversity, sex differences, economic, and ethical challenges. Till now the genetic risk predictions have had no added value to prediction models for T2DM based on simple clinical risk factors, provided that GRS have been applied to the “general” population (Meigs et al., 2008). There is a shift in the understanding with a key element that rather the principal disease traits (i.e., sub-phenotypes or “endophenotypes”) should be put into the focus of the genetic prediction models than the “general population” to gain success in the clinical application of GRS. It is estimated that only 30% of (T2DM) patients might fall within the top 10% of a disease trait (“cluster”) associated GRS (Florez, 2019). Therefore, we may conclude that significant associations and the added value of the genetic prediction models may remain hidden with the “usual approach” when the analysis is only applied to less narrow, more heterogenous, “too general” populations, instead of assessing disease subtypes or key traits referring to specific mechanisms. Others also concluded that T2DM genetic risk score is not sufficient to improve health or health-economic, rather the better identification of metabolic sub-phenotypes with stronger genetic background might improve the opportunities for health translation (Meigs, 2019).

Few authors already argue that it is essential to stratify people based on genomic DNA variation and for some common diseases, including coronary artery disease and atrial fibrillation, PRSs reached to a point when it is appropriate to implement into clinical care (Khera et al., 2018). In addition to PRS studies that are designed to identify high-risk groups recently there is also an intriguing discussion about the potential value for the use in evaluating therapeutic response (Gibson, 2019). Nevertheless, the same reviews are also outlining that when PR scores identify the upper two deciles of the risk distribution it is on the cost of declining sensitivity. PRS sensitivity (defined as the approximate proportion of cases captured by the PRS at a given PRS percentile) usually drops below 50% in such typical cases as estimated from a PRS created from many thousands of variants in a population of hundreds of thousands of individuals, such as the UK Biobank (Gibson, 2019).

## GWAS Findings as “Clinically Crude Data”

From the clinician's perspective the genetic findings of GWASs might be interpreted as “crude” data. A refined disease subgroup definition and a more precise phenotyping might be applied to get closer to a more meaningful clinical association (Ahlqvist et al., 2018; Meigs, 2019). We highlight that in case of a few—already identified disease associated—gene variants the genetic effect sizes may be substantially higher provided that we assess more precisely defined clinical traits and treatment modalities. This approach that might potentially be applicable only for a few gene variants, but could help more in the clinical use of genetic information.

## Association of *MTNR1B* rs10830963 Gene Variant and Diabetes Mellitus Traits

### Type 2 Diabetes Mellitus (T2DM) Development

The *MTNR1B* rs10830963 associated effects on non-autoimmune diabetes mellitus (DM) traits are discussed as an example, more specifically the differences in the genetic effect sizes for T2DM development and GDM development/therapy.

The odds ratios (ORs)—referring to the genetic effect sizes—associated to the rs10830963 variant are very moderate for T2DM development (1.09–1.12-fold per *G* allele) (Lyssenko et al., 2009; Prokopenko et al., 2009).

### Gestational Diabetes Mellitus (GDM) Development

In contrast, independent case-control genetic studies report OR values between 1.29 and 2. for GDM development in Middle European (Vejrazkova et al., 2014; Rosta et al., 2017), Mediterranean Hispanic (García de la Torre et al., 2019), Middle Eastern (Alharbi et al., 2019) Asian (Wu et al., 2016) and Russian (Popova et al., 2017) pregnant populations. The *MTNR1B* rs10830963 associated effects appeared to be more preponderant provided that the mean pre-pregnancy BMI was above 25 kg/m^2^ (Wu et al., 2016). Furthermore the OR values for GDM development were particularly high (1.96) when BMI was above 30 kg/m^2^ (Alharbi et al., 2019) even after adjustment to BMI. This indicates substantially higher *MTNR1B* rs10830963 associated genetic effect sizes when GDM compared to T2DM development and therefore suggests additional pregnancy specific factors.

## Association of *MTNR1B* rs10830963 Gene Variant and Prevention and Therapy of GDM

We found that the genetic effect sizes are further increased (OR = 5.2 for *G* allele carriers) on the need for antenatal insulin therapy (AIT) initiation in Hungarian GDM patients (Firneisz et al., 2018) above the pre-pregnancy BMI threshold of 29 kg/m^2^. Furthermore, it was independently reported (Grotenfelt et al., 2016) that the rs10830963 *G* risk allele carrying significantly decreased the odds with a similar effect size (OR for intervention failure in *G* allele carriers >5) of responding to an early (from the 13th gestational week) medical nutrition therapy and lifestyle intervention in high-risk (pre-pregnancy BMI ≥ 30 kg/m^2^ and/or prior GDM) Finnish pregnant women. In addition the *MTNR1B* rs10830963 variant is reported to augment the effect of lifestyle risk factors and dietary habits (in particular sausage consumption) on GDM development (Popova et al., 2017).

These three reports independently indicate clinical consequences associated to the *MTNR1B* rs10830963 risk genotype in the prevention strategies and also the treatment of high risk disease subpopulations.

## Difference in Genetic Effect Sizes and Potential Explanations

**Figure 1:**
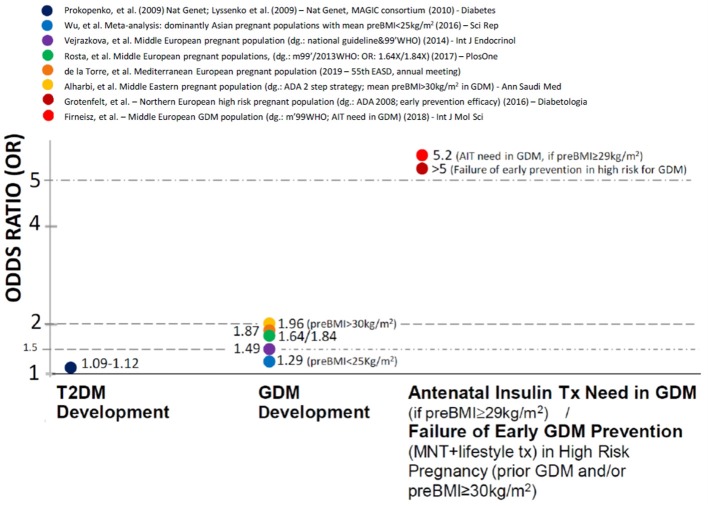
The association of *MTNR1B* rs10830963 gene variant with different clinical traits in non-autoimmune diabetes mellitus and the corresponding odds ratios.

The high effect sizes in precisely identified patient sub-populations might be potentially useful for guiding the clinical practice, although it might be relevant for only around 30% of the entire disease population in T2DM (Florez, 2019). A novel approach of associating clinical utility to the crude GWAS findings and the *MTNR1B rs10830963* associated genetic effects to distinct endophenotypes within the diabetes mellitus spectrum might demonstrate consistently similar features (Figure 1).

One possible pregnancy specific factor to explain this phenomenon might be the robust increase (>3x at the end of pregnancy compared to levels before the 24th gestational week) in the night time maternal serum melatonin (physiological ligand of the *MTNR1B* encoded MT2 receptor) concentrations (Nakamura et al., 2001). The continuous rise of the night time maternal serum melatonin levels from the 24 to 28th gestational week accords with the time-frame of the routine diagnostic OGTT during pregnancy. The expression of *MTNR1B* mRNA is generally very low in all tissues, yet a higher expression (HPA RNA-seq normal tissues) is found in the placenta where it is also expressed at protein level (MT2) (Li et al., 2018). One may hypothesize that the exceptionally high maternal ligand concentrations during the 3rd trimester are somehow causally related to the multiplied effect sizes associated to the rs1083096 receptor gene variant in GDM development and treatment.

In addition, the difference in age ranges of the study participants in T2DM GWAS (35–70 years of age) vs. GDM genetic association studies (substantially younger, reproductive age) might also contribute to the attenuated *MTNR1B* rs10830963 associated genetic effect sizes reported for T2DM compared to GDM development. This could potentially be due to the age-associated decline in melatonin production and melatonin receptor expression (Hill et al., 2011) and the ab ovo disrupted central clock driven circadian melatonin output in metabolic diseases in the elderly (Belancio et al., 2014).

If we systematically narrow the studied population to precisely defined subgroups (T2DM development < GDM development < AIT initiation in GDM with BMI over 29 kg/m^2^), we experience a step-wise increase in the genetic effect size (Figure 1).

## The Potential Significance of *MTNR1B* rs10830963 in GDM Complications

Provided that the observation is approached reversely, from the point of genetic associations to the complications in populations with high enough proportion of undiagnosed/untreated GDM patients (“natural disease course”) then we should—in theory—arrive back to the *MTNR1B* rs10830963 variant. Recently, a large GWAS reported maternal genetic effects for the neonatal birth weight that were independent of offspring genetics (Beaumont et al., 2018). Among the identified maternal signals the *MTNR1B* rs10830963 was characterized by the lowest *p*-value association with neonatal birthweight and had the highest genetic effect size out of the more than 8 million gene variants assessed in the united EGG and UKBB meta-analysis. It is meaningful that this study population was unadjusted to glycemia during pregnancy/presence of GDM (which was not systematically excluded using an OGTT during pregnancy), despite these maternal conditions are well-known to be associated with offspring birth weight. We should hypothesize that this birth weight increasing genetic effect also occurs when the neonatal outcome is expressed in birth weight percentiles and therefore the maternal *MTNR1B* rs10830963 might be associated with higher occurrence of large for gestational age (LGA) one of the most known GDM complications.

## Discussion

The wider clinical use of genetic data in complex diseases could be determined by

- The risk allele frequency (RAF) in the population,
- The genetic effect size related to a clinically significant outcome
- And the appropriate definition of the sub-population (“endophenotype”) in which the prediction of the therapeutic response is aimed.

The proportion of *MTNR1B* rs10830963 risk *G* allele carriers reached/exceeded the 50% in the populations reported on Figure 1 that might indicate a rationale for population based screening programs of high risk GDM pregnancies in these regions. Our response as clinicians should already be triggered in any case when a meaningful outcome, such as antenatal insulin treatment in GDM is assessed in a precisely defined clinical trait and the exposure results in an OR value over 5, even in the case of a single risk gene variant without the use of a challenging PRS/PHS. AIT need in GDM might still be a complex trait from the genetic point, however we think that—unlike T2DM development—this genetic complexity could be characterized by a gene variant with the major effect (*MTNR1B* rs10830963) in populations where it occurs with high enough MAF (such as European, Asian and Middle-Eastern origins).

Interestingly, in parallel with identifying the upper two deciles in the risk distribution in this case this model employing a single, but high effect gene variant could still keep the pick-up rate (equivalents to the sensitivity) above 50%, in contrast to PRS which—in general—still faces the problem of declining sensitivity (defined as the approximate proportion of cases captured by the PRS at a given percentile) as the cost that the higher precision and OR values could only be gained with increased number of gene variants (Gibson, 2019).

We may conclude that an approach employing even a single gene variant might potentially be more informative in predicting the therapeutic responses provided that this model is used in a narrow, clinically well-defined “endophenotype” in patients already diagnosed with a prevalent disease if the genetic effect size is high enough and the variant is not rare.

However, we suspect that such clinical conditions might not be that frequent and therefore the described approach could be considered as auxiliary to the PRS which appears to be more of clinical use (even if it is currently limited) when the aim is to assess the genetic risk in largely different, broader, more heterogeneous populations with individually weak effect gene variants.

Three independent genetic studies confirmed that the maternal *MTNR1B* rs10830963 risk variant is consistently associated with major forms of clinical consequences (Florez, 2019), such as

- Early *prevention* failure in high risk pregnant (Grotenfelt et al., 2016)
- *Therapy* (AIT need) in high risk GDM (Firneisz et al., 2018) subpopulations and
- A neonatal c*omplication* related trait in the general population (Beaumont et al., 2018).

Therefore we may not only hypothesize that the precision medicine approach (employing the *MTNR1B* rs10830963 gene variant) in predicting the AIT need in a narrow subpopulation of patients already diagnosed with GDM might result in better and earlier identification of patients who would require antenatal insulin therapy (Firneisz et al., 2018), but also—consistently with the findings of the maternal GWAS on neonatal birthweight (Beaumont et al., 2018)—we may potentially expect the reduction of complications (e.g., macrosomia/LGA rate).

Nevertheless, as an ultimate gold standard first step in clinics an RCT should be conducted to confirm that this genetic precision medicine based approach might result in any of the expected clinical benefits at all.

## Author Contributions

GF conceived the opinion and wrote the manuscript. KR, JR, JH, AK-W, AS, and ÁN reviewed the manuscript.

## Conflict of Interest

The authors declare that the research was conducted in the absence of any commercial or financial relationships that could be construed as a potential conflict of interest.
